# Anandamide prevents the adhesion of filamentous *Candida albicans* to cervical epithelial cells

**DOI:** 10.1038/s41598-020-70650-6

**Published:** 2020-08-13

**Authors:** Ronit Vogt Sionov, Mark Feldman, Reem Smoum, Raphael Mechoulam, Doron Steinberg

**Affiliations:** 1 Biofilm Research Laboratory, The Faculty of Dental Medicine, The Hebrew University of Jerusalem, Jerusalem, Israel; 2 The Faculty of Medicine, The Institute for Drug Research, The Hebrew University of Jerusalem, Jerusalem, Israel

**Keywords:** Antimicrobials, Fungi, Experimental models of disease

## Abstract

Candidiasis is a fungal infection caused by *Candida* species that have formed a biofilm on epithelial linings of the body. The most frequently affected areas include the vagina, oral cavity and the intestine. In severe cases, the fungi penetrate the epithelium and cause systemic infections. One approach to combat candidiasis is to prevent the adhesion of the fungal hyphae to the epithelium. Here we demonstrate that the endocannabinoid anandamide (AEA) and the endocannabinoid-like *N*-arachidonoyl serine (AraS) strongly prevent the adherence of *C. albicans* hyphae to cervical epithelial cells, while the endocannabinoid 2-arachidonoylglycerol (2-AG) has only a minor inhibitory effect. In addition, we observed that both AEA and AraS prevent the yeast-hypha transition and perturb hyphal growth. Real-time PCR analysis showed that AEA represses the expression of the *HWP1* and *ALS3* adhesins involved in *Candida* adhesion to epithelial cells and the *HGC1*, *RAS1*, *EFG1* and *ZAP1* regulators of hyphal morphogenesis and cell adherence. On the other hand, AEA increased the expression of *NRG1*, a transcriptional repressor of filamentous growth. Altogether, our data show that AEA and AraS have potential anti-fungal activities by inhibiting hyphal growth and preventing hyphal adherence to epithelial cells.

## Introduction

*Candida albicans* is a common commensal organism in the genitourinary tracts, the intestine and the oral cavity, but can also be pathogenic causing infections by invading and damaging epithelial cells, a condition called candidiasis. In addition, *C. albicans* can cause life-threatening systemic infections by penetrating through the epithelial barriers^1^. *C. albicans* is a dimorphic fungus that can transform from budding yeast form cells at room temperature to invasive filamentous hyphae at 37 °C, a process vital to pathogenesis^2^. This transition is tightly regulated. The mitogen-activated protein kinase Mkc1 that is activated upon physical contact, is required for invasive hyphal growth and normal biofilm development^3^. The transcription factor Czf1 controls the contact-dependent invasive filamentation^4^. Other gene products regulating filamentous growth include the transcriptional regulators Cph1, Ume6, Bcr1, Tec1 and Efg1^5^. Hyphal growth is characterized by the expression of different genes, some of which are involved in adhesion.

Biofilm formation by *C. albicans* is characterized by four major phases: adherence of yeast cells to a surface; initiation of biofilm formation where the hyphae are formed; maturation into complex, structured biofilm in which the fungi are embedded in an extracellular matrix; and dispersion of yeast cells from the biofilm to initiate biofilms at other sites^5^. The dispersion from biofilms may lead to systemic infections in the bloodstream and dissemination into other tissues. More than 50 interconnected transcriptional regulators are involved in regulating biofilm formation^5^. Initial attachment of *C. albicans* to a surface appears to involve the glycosylphosphatidylinositol (GPI)-linked cell wall protein Eap1 and the agglutinin-like protein Als1^6^. The agglutinin-like protein Als3 and the hyphal-specific wall protein-1 gene product Hwp1 function as complementary adhesins involved in cell–cell and cell-surface interactions of hyphae^2,6^. Als3 was found to bind N-Cadherin on endothelial cells and E-Cadherin on epithelial cells^7^. Strains defective in *ALS3* can form mycelium normally, but are defective in biofilm formation^8^. *HWP1* encodes a cell wall mannose protein essential for normal growth of the mycelium^2^. *HWP1* mutant strains could not stably adhere to the epithelial mucosal cells and were more easily engulfed and cleaned by the host cells^9^. *ALS3*, *HWP1*, *HGC1* and *ECE1* are upregulated in hyphae^2^. Hyphal G cyclin 1 (Hgc1) is involved in regulating mycelial growth and represses cell separation from hyphae^2^. *ECE1* encodes for candidalysin, a peptide toxin that activates epithelial cells^10^ and leads to cytolysis of mononuclear phagocytes^11^, and as such is considered to be a virulence factor. *EED1* and *PGA34* are dispensable for epithelial invasion, but essential for damage of epithelial cells^12^.

One approach to prevent systemic candidiasis is to prevent the adherence of the filamentous fungi to epithelial cells. Here we have studied the ability of the endocannabinoids anandamide (*N*-arachidonoylethanolamine; AEA) and 2-arachidonoylglycerol (2-AG), and the endocannabinoid-like compound N-arachidonoyl serine (AraS) to prevent the interaction between *C. albicans* hyphae and the epithelial cells. Endocannabinoids are endogenous bioactive lipids derived from arachidonic acid which is produced by hydrolysis of membrane phospholipids^13^. The endocannabinoid system affects multiple functions including feeding, pain, learning and memory^14^. In addition, AEA has been shown to exert anti-inflammatory activities attenuating the development of inflammation in a mouse model of ulcerative colitis^15^. In human and rodents, AEA acts as an endogenous agonist of the cannabinoid CB1 and CB2 receptors, and can also activate the vanilloid receptor TRPV1 resulting in transient calcium influx^16,17^. Also 2-AG may act on other receptors besides CB1 and CB2, including GABA_A_, PPARγ, TRPV1 and GPR55^18^. AraS binds weakly to the CB1, CB2 and TRPV1 receptors^17^, but seems to act on GRP55 to stimulate angiogenesis and endothelial wound healing^19^.

We have previously shown that both AEA and AraS reduce biofilm formation of methicillin-resistant *S. aureus* (MRSA)^20^ and sensitize MRSA to antibiotics^21^. Both compounds reduced the metabolic activity and spreading ability of these bacteria^20^. So far, endocannabinoids have not been tested for their activity on *Candida*, and the aim of the present research was to study this issue. We show here that treatment of *C. albicans* with either AEA or AraS strongly reduced the interaction between *C. albicans* hyphae and cervical epithelial cells. In addition, we observed that AEA and AraS prevented yeast-hypha transition and the growth of preformed hyphae. Gene expression studies showed down-regulation of the adhesins *HWP1* and *ALS3*, the transcriptional regulators *EFG1* and *ZAP1*, and the hyphal morphogenesis regulators *HGC1* and *RAS1*. On the other hand, *NRG1*, a repressor of filamentous growth, was upregulated.

## Results

### Anandamide and N-arachidonoyl serine prevent the yeast-hypha transition of *Candida albicans*

**Figure 1:**
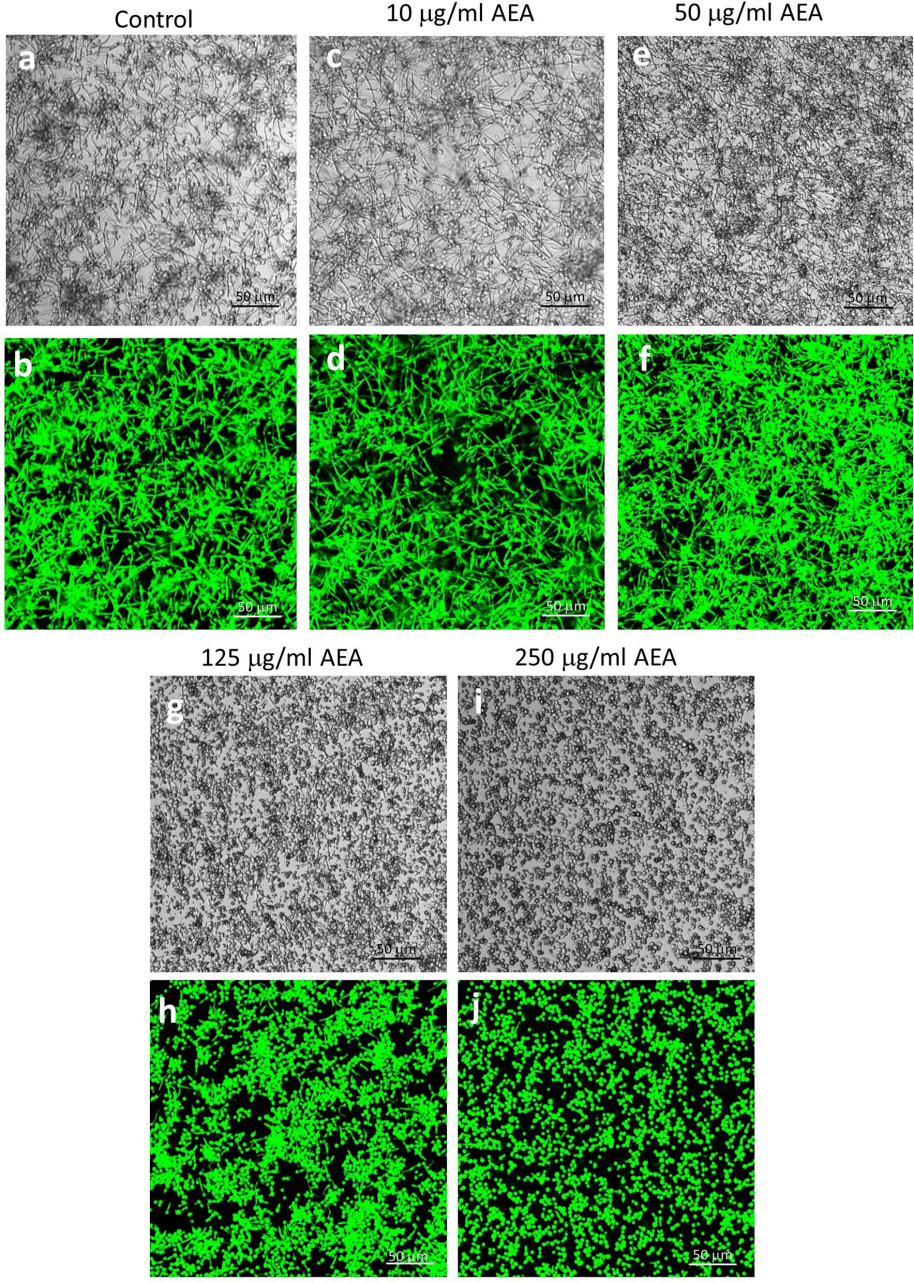
Anandamide prevents yeast-hypha transition. GFP-expressing *Candida albicans* taken from PDA plates in its yeast form was incubated with different concentrations of AEA at 37 °C for 4 h, and the morphology studied by confocal microscopy. (**a**, **c**, **e**, **g**, **i**) Bright field. (**b**, **d**, **f**, **h**, **j**) green fluorescence.

It is well known that the virulence of *Candida* depends on its transition from the yeast form to filamentous hyphae^2^*.* It was therefore important to study the effect of the endocannabinoids anandamide (AEA) and 2-arachidonoylglycerol (2-AG), and the endocannabinoid-like *N*-arachidonoyl serine (AraS) on this transition. To this end, we exposed GFP-expressing *Candida albicans* in the yeast form to various concentrations of AEA, AraS and 2-AG and incubated them for 4 h at 37 °C, a condition resulting in the transition to hyphae. Most of the fungi have transformed to filamentous hyphae in the control and in the fungi exposed to 10 μg/ml AEA (Fig. 1a–d). In samples treated with 50 μg/ml AEA, most of the fungi had transformed to hyphae, but also several fungi in yeast form and fungi with short hyphae were seen (Fig. 1e–f). However, almost no transformation to hyphae was observed in *Candida* exposed to 125 and 250 μg/ml AEA (Fig. 1g–j), indicating that AEA at these concentrations prevents the yeast-hypha transition. Similarly, AraS strongly inhibited yeast-hypha transition at 125 and 250 μg/ml, with partial inhibitory effect at 50 μg/ml (Suppl. Figure 1). 2-AG had no significant effect on the yeast-hypha transition (data not shown). When looking at the vegetative growth of *C. albicans* in the yeast form, a delay in the growth was observed in the presence of higher concentrations of AEA, AraS and 2-AG (Suppl. Figure 2).

**Figure 2:**
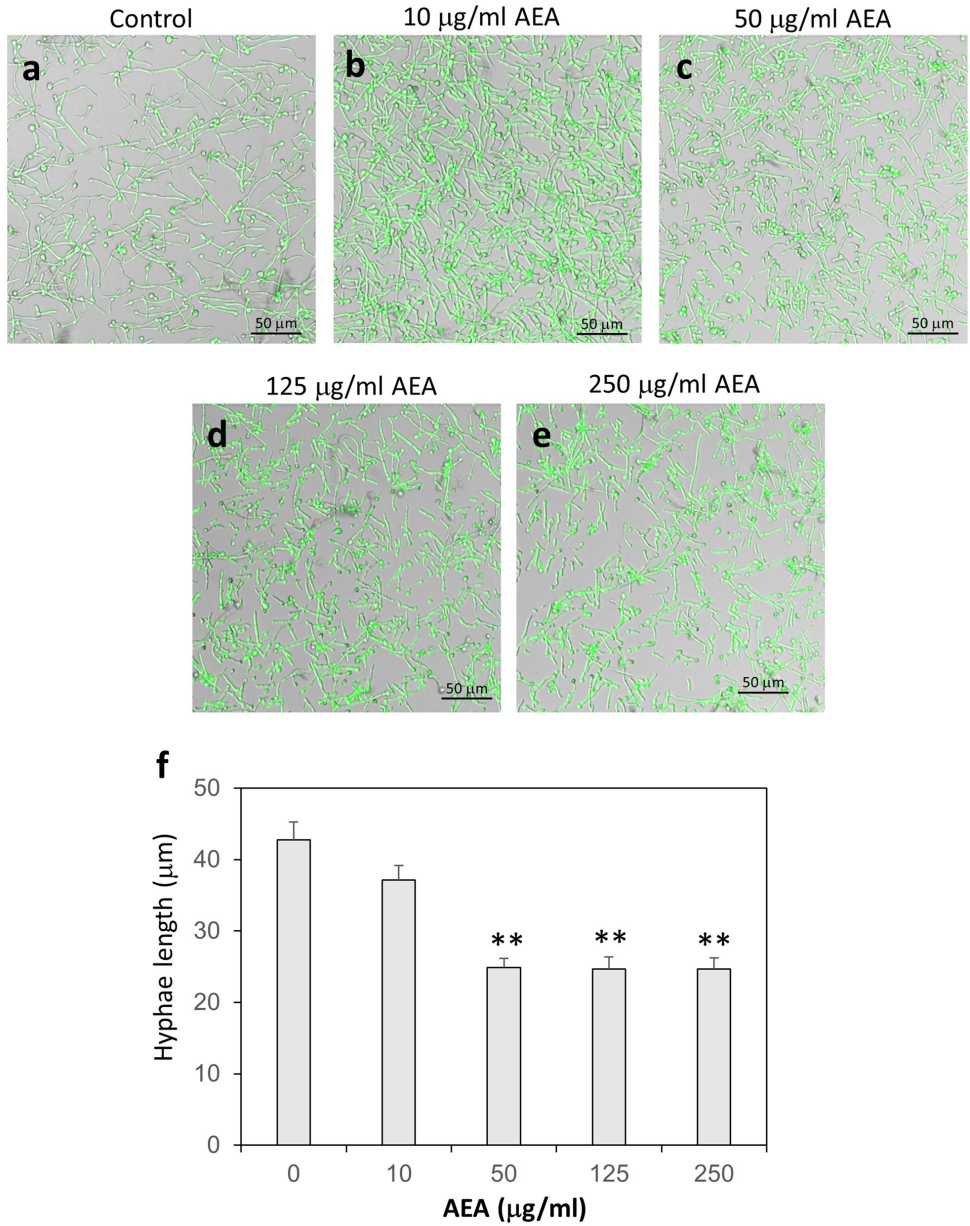
AEA treatment of *C. albicans* hyphae leads to shorter hyphae than control. (**a**–**e**) Confocal microscopy of control and AEA-treated *C. albicans.* GFP-expressing *C. albicans* taken from PDA plates in its yeast form was incubated in RPMI at 37 °C for 16 h, and then exposed to different concentrations of AEA for 1 h and the morphology studied by confocal microscopy. The merged bright fields and green fluorescence are shown. (**f**) The average hyphae lengths of untreated and AEA-treated *C. albicans*. Number of hyphae measured for each sample was 90–110 from 4–5 different fields. **p < 0.001.

### Anandamide impairs the further growth of preformed hyphae

**Figure 3:**
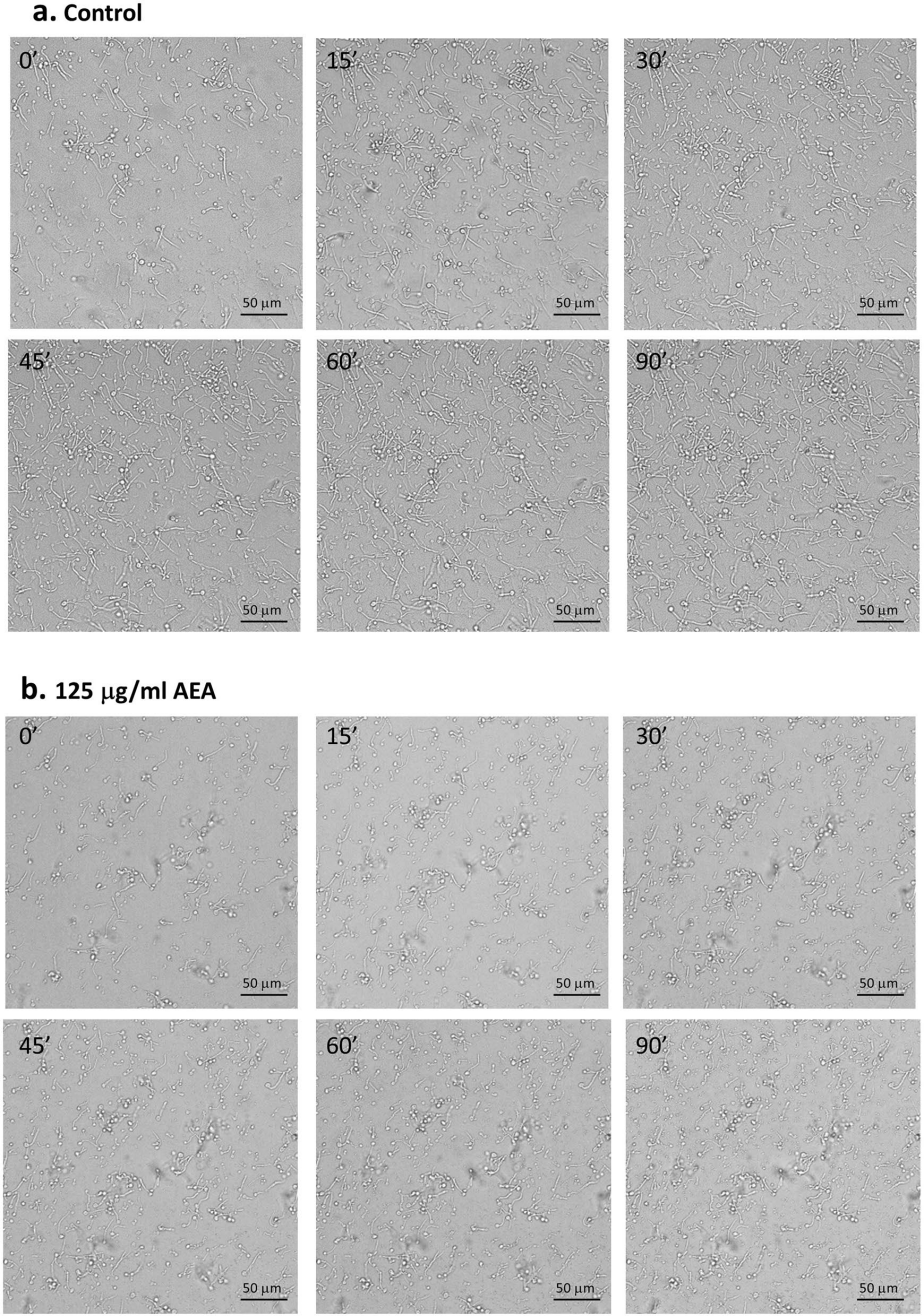
AEA inhibits hyphal growth. (**a**) Spinning scan microscopy of control hyphae at time 0, 15, 30, 45, 60 and 90 min. (**b**) Spinning scan microscopy of hyphae at time 0, 15, 30, 45, 60 and 90 min in the presence of 125 μg/ml AEA. *C. albicans* in yeast form was allowed to form hyphae by incubating them 4 h at 37 °C, and thereafter a time-lapse study was performed using a Nikon spinning scan microscopy in the absence (**a**) or presence of 125 μg/ml AEA (**b**). Time 0 is 30 min after adding AEA.

Since AEA prevented the morphogenetic switch from yeast to hyphae, we wondered whether AEA could affect the hyphae after being formed. For this purpose, we allowed the *Candida* to form hyphae by an overnight incubation at 37 °C, and then exposed the hyphae to various concentrations of AEA for 1 h. The majority of the hyphae length in the control and 10 μg/ml AEA-treated samples ranged between 25–60 μm (Fig. 2a,b). Most of the *C. albicans* hyphae treated with 50, 125 and 250 μg/ml AEA for 1 h showed shorter hyphae length ranging from 15–30 μm (Fig. 2c–e). Occasionally, some hyphae with exceptional long length (90–100 μm) appeared in both the control and treated samples (Fig. 2). We next wanted to know whether the shorter hyphae observed in the AEA-treated samples are due to an inhibition of hyphal growth. To study this possibility, *C. albicans* in its yeast form was first allowed to form hyphae by incubating them 4 h at 37 °C, and then subjected to a 3 h time-lapse microscopy at 37 °C in the absence or presence of 125 μg/ml AEA. As expected, the hyphae continued to grow in the control samples (Fig. 3a and Suppl. Figure 3a—time-lapse video). However, most of the hyphae ceased growing after being exposed to AEA (Fig. 3b and Suppl. Figure 3b—time-lapse video), suggesting that AEA interferes with hyphal morphogenesis. Similar inhibition of hyphal growth was observed when exposing the hyphae to 50 μg/ml AEA (data not shown).

### Anandamide (AEA) prevents the adherence of *Candida albicans* to cervical epithelial cells

**Figure 4:**
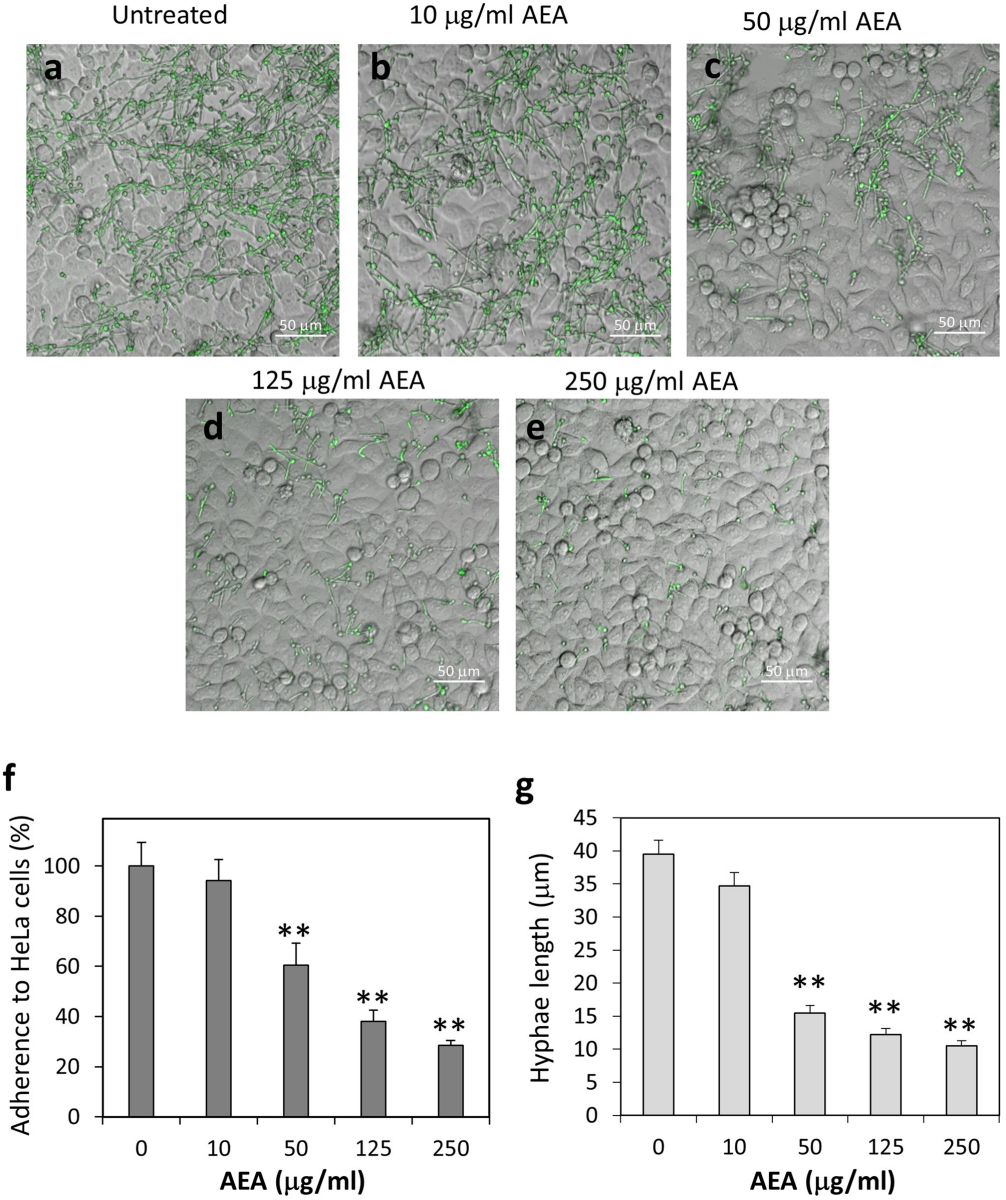
AEA treatment of *C. albicans* reduced their adherence to HeLa cervical epithelial cells. (**a–e**) Confocal microscopy of co-cultures of control and AEA-treated *C. albicans* on confluent HeLa cells. The fungi were pretreated with the indicated concentrations of AEA for 1 h prior to co-incubation with HeLa cells for an additional 1 h. The fungi express GFP. (**f**) Quantification of the relative adherence of untreated and AEA-treated *C. albicans* to HeLa cells. 8–10 images taken from 3 independent experiments were analyzed for each treatment. (**g**) The average hyphae lengths of untreated and AEA-treated *C. albicans* that were able to adhere to HeLa cells. The number of hyphae measured for each treatment was 50–75. **p < 0.001.

**Figure 5:**
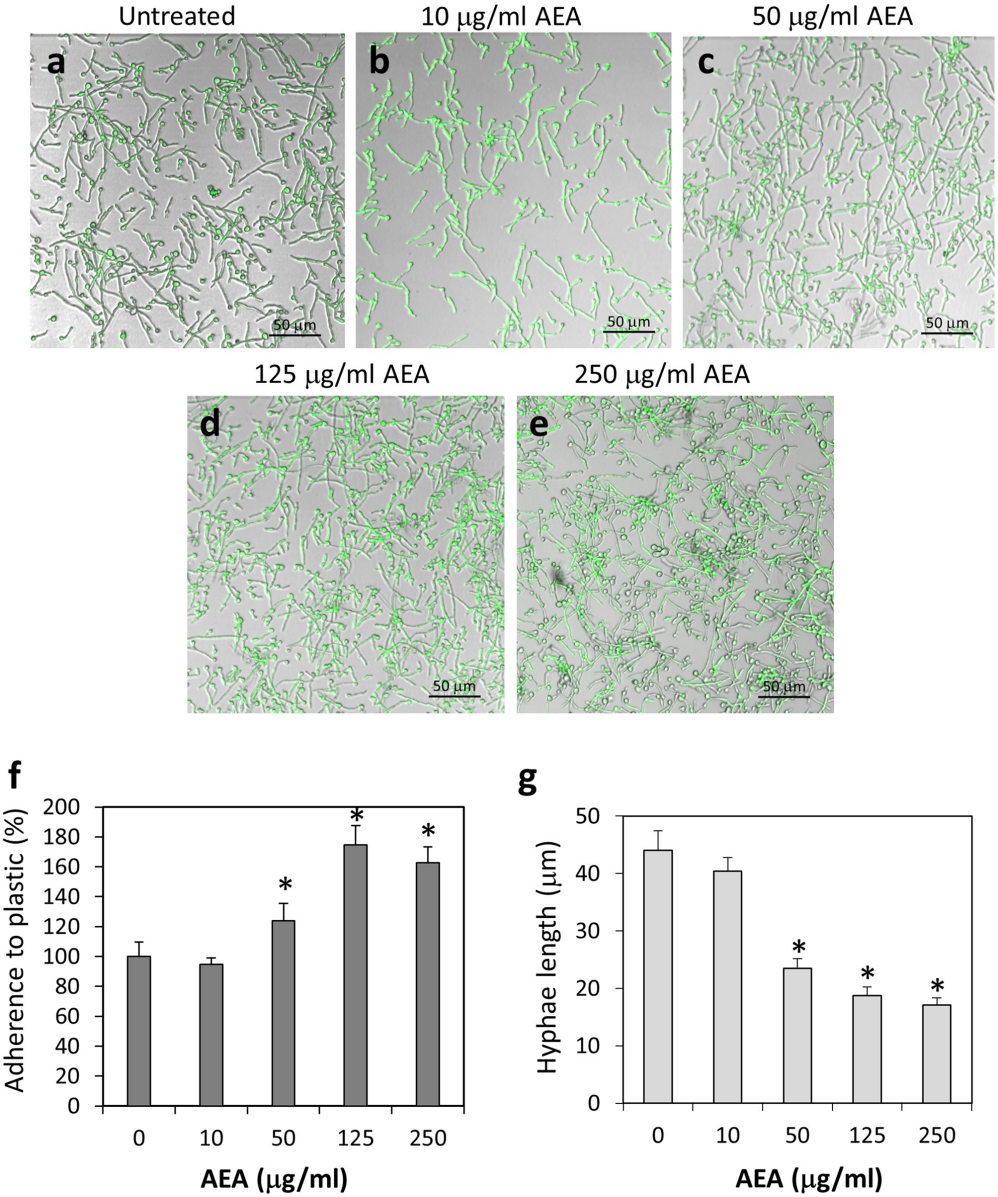
*C. albicans* treated with AEA adhered to polystyrene plastic surface even better than untreated fungi. (**a–e**) Confocal microscopy of control and AEA-treated *C. albicans* on tissue culture plates. The fungi were pretreated with the indicated concentrations of AEA for 1 h prior to incubation on polystyrene surfaces for an additional 1 h. (**f**) Quantification of the relative adherence of untreated and AEA-treated *C. albicans* to plastic. 8–10 images taken from 3 independent experiments were analyzed for each treatment. (**g**) The hyphae lengths of untreated and AEA-treated *C. albicans* that were able to adhere to plastic. The number of hyphae measured for each treatment was 50–75. *p < 0.05.

Next we studied the effect of AEA on *C. albicans* adherence to cervical epithelial cells. GFP-expressing *C. albicans* were allowed to form hyphae by an overnight incubation at 37 °C. The hyphae were pretreated with various concentrations of AEA for 1 h, and then co-cultured on confluent HeLa cervical epithelial cells for another hour (Fig. 4a–e). In parallel, the same fungi samples were incubated on tissue culture plastic plates as controls that reflect the inputs (Fig. 5a–e). The morphology of the whole fungal population prior to incubation with HeLa or on plastic is shown in Fig. 2. Pretreatment of *C. albicans* hyphae with 50 μg/ml and 125 μg/ml AEA (Fig. 4c,d) reduced their adherence to the epithelial cells by 40 ± 8% and 62 ± 4%, respectively, with a statistical significance of *p* < 0.001 compared to control (Fig. 4f). Increasing the AEA concentration to 250 μg/ml (Fig. 4e) did only cause a slightly higher inhibition of 72 ± 2% (Fig. 4f), suggesting that a plateau effect is observed at 125 μg/ml. Of note, AEA-treated *C. albicans* hyphae that were able to bind to the epithelial cells, showed 3–fivefold shorter hyphae (Fig. 4d,e) compared to control fungi (Fig. 4a) with a *p* < 0.001 for 50–250 μg/ml AEA (Fig. 4g). A maximal effect on hyphae length was observed at 50 μg/ml (Fig. 4g). At a concentration of 10 μg/ml, AEA had no significant effect on the hyphae length (Fig. 4b). The hyphae that adhered to HeLa cells were also thinner and several fungi appeared without hyphae at all when using 50–250 μg/ml AEA. The appearance of shorter hyphae adherent to HeLa cells following AEA treatment is a direct consequence of the AEA effect on hyphal growth as described above (Figs. 2, 3). Of particular importance is the preferential inhibition of hyphal adhesion to HeLa cells in comparison to plastic as shown by the relative reduction in the ratio of HeLa-adherent versus plastic-adherent hyphae (Suppl. Figure 4). Pretreatment of HeLa cells with AEA prior to addition of *C. albicans* didn't alter the attachment of the fungi to the cells (data not shown). It should be noted that normal untreated hyphae of different lengths exhibited similar ability to adhere to HeLa cells (Suppl. Figure 5), meaning that the preferential appearance of shorter adherent hyphae after exposure to AEA is a direct consequence of the treatment.

In contrast to the reduced adherence of AEA-treated *C. albicans* to HeLa cells, the AEA-treated fungi adhered even better to polystyrene tissue culture plates in comparison to untreated fungi (Fig. 5a–e) with statistical significance in the concentration range of 50–250 μg/ml AEA (Fig. 5f; *p* < 0.05). This accords with the higher amount of hyphae observed in AEA-treated samples in comparison to control samples (Fig. 5). The hyphae length of AEA-treated *C. albicans* that bound to plastic were about twofold shorter on average in comparison to control (Fig. 5g) with statistical significance in the concentration range of 50–250 μg/ml AEA (*p* < 0.05). When incubating the *C. albicans* with AEA for 24 h, there was no significant alteration in the biofilm mass formed on plastic (Suppl. Figure 6). However, both 2-AG and AraS significantly reduced the biofilm mass on plastic in a dose-dependent manner with a *p* < 0.05 (Suppl. Figure 6).

**Figure 6:**
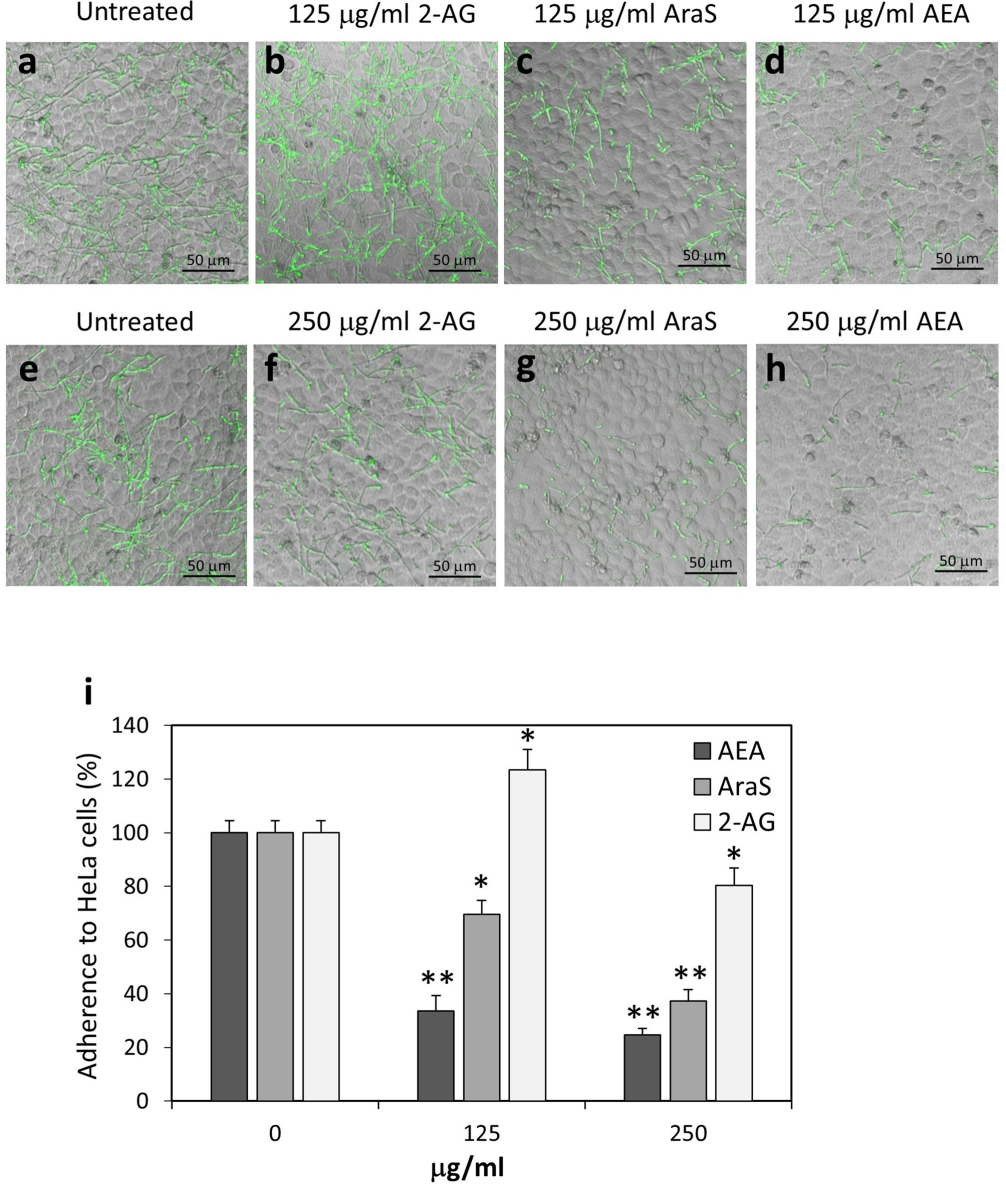
*C. albicans* treated with AraS showed strong reduction in their adherence to cervical epithelial cells. (**a**–**h**) Confocal microscopy of co-cultures of control and AEA-treated *C. albicans* on confluent HeLa cells. The fungi were pretreated with the indicated concentrations of 2-AG, AraS or AEA for 1 h prior to co-incubation with HeLa cells for an additional 1 h. (**i**) Quantification of the relative adherence of untreated and treated *C. albicans* to HeLa cells. 5–7 images were analyzed for each treatment. *p < 0.05, **p < 0.001.

### N-Arachidonoyl serine (AraS), but not 2-arachidonoylglycerol (2-AG), prevents the adherence of *Candida albicans* to cervical epithelial cells

**Figure 7:**
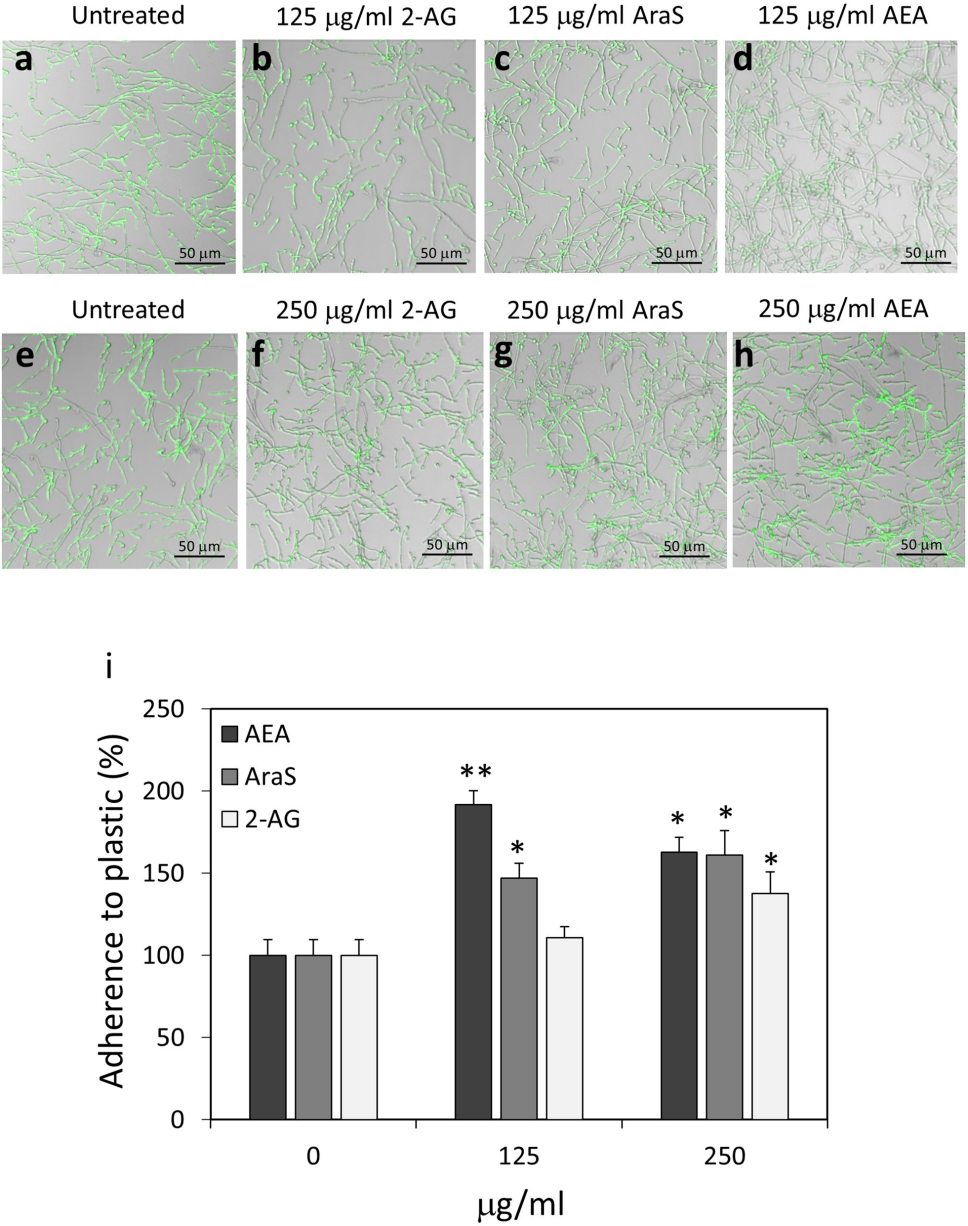
*C. albicans* treated with 2-AG, AraS or AEA retained binding capacity to polystyrene surface. (**a**–**h**) Confocal microscopy of control and treated *C. albicans* bound to polystyrene plastic surface. The fungi were pretreated with the indicated concentrations of 2-AG, AraS or AEA for 1 h prior to incubation in tissue culture plates for an additional 1 h. (**i**) Quantification of the relative adherence of untreated and treated *C. albicans* to plastic. 5–7 images were analyzed for each treatment. *p < 0.05, **p < 0.001.

We next analyzed the effect of AraS and 2-AG on the ability of *C. albicans* to adhere to cervical epithelial cells and compared their effect with that of AEA (Fig. 6a–h). Pretreatment of *C. albicans* with 125 μg/ml and 250 μg/ml AraS for 1 h (Fig. 6c,g) reduced adherence to the epithelial cells by 30 ± 5% and 63 ± 5%, respectively, with a statistical significance of *p* < 0.05 compared to control (Fig. 6i). The hyphae of the fungi that adhered to the epithelial cells following AraS treatment (Fig. 6g) were shorter and thinner in comparison to untreated fungi (Fig. 6a,e). However, treatment of *C. albicans* with 125 μg/ml and 250 μg/ml 2-AG (Fig. 6b,f) didn’t interfere with their adherence to HeLa cells (Fig. 6i). Both AraS and 2-AG treated *C. albicans* adhered well to polystyrene plastic within 1 h (Fig. 7). This is in contrast to the reduced biofilm mass formation on plastic after 24 h incubation in the presence of the compounds (Suppl. Figure 6).

### AEA altered the expression of genes involved in adhesion and hyphal morphogenesis

In order to understand the anti-adhesive effects of AEA on *C. albicans* interaction with epithelial cells, we exposed the fungi to various concentrations of AEA for 2 h, followed by gene expression analysis of genes relevant for biofilm formation, adherence and hyphal morphogenesis (Tables 1, 2 and 3). Some genes were upregulated including the *ALS1* adhesion molecule, the transcription factor *TEC1* involved in hyphal development and the transcriptional repressor *NRG1* that prevents filamentous growth (Table 1). *ALS1* and *TEC1* were mainly upregulated at the lower concentrations (10 and 50 μg/ml AEA), while *NRG1* was upregulated at the higher concentrations (50–250 μg/ml AEA). Of note, the three multidrug efflux transporters *MDR1*, *CDR1* and *CDR2* were strongly upregulated (Table 1). On the other hand, AEA repressed the expression of the adhesins *HWP1* and *ALS3*, the cell elongation protein *ECE1*, the signal transduction regulators *HGC1* and *RAS1* and the transcription regulators *EFG1* and *ZAP1* (Table 2). Also the cell hydrophobicity-associated protein *CSH1* was strongly repressed as well as the virulence factor phospholipase D1 (*PLD1*) (Table 2). There were also several genes that were not significantly affected by AEA including the cell wall adhesion *EAP1* and the anti-adhesive protein *YWP1* (Table 3). Altogether, these alterations in gene expression may explain, at least in part, the inhibition of hyphal growth by AEA and the reduced adherence of AEA-treated hyphae to epithelial cells.

**Table 1:** Genes that are upregulated in *C. albicans* after a 2 h-incubation with AEA.

Gene	AEA conc (μg/ml)	Fold change	Function
*ALS1*	10	+ 1.9 ± 0.5*	Major cell surface adhesion protein which mediates yeast-to-host tissue adherence and yeast aggregation
	50	+ 4.1 ± 1.3*	
	125	n.s	
	250	n.s	
*TEC1*	10	+ 2.1 ± 0.4*	Transcription factor of the TEA/ATTS family which regulates genes involved in hyphal development, cell adhesion, biofilm development, and virulence
	50	+ 3.9 ± 1.3*	
	125	n.s	
	250	n.s	
*NRG1*	10	n.s	Transcriptional repressor of filamentous growth
	50	+ 2.2 ± 0.7*	
	125	+ 2.6 ± 1.0*	
	250	+ 2.0 ± 0.3*	
*MDR1*	10	− 3.0 ± 0.3*	Multidrug resistance protein 1
	50	n.s	
	125	+ 2.5 ± 1.1*	
	250	n.s	
*CDR1*	10	+ 3.0 ± 0.3*	Pleiotropic ABC efflux transporter of multiple drugs
	50	+ 4.8 ± 1.6*	
	125	+ 1.6 ± 0.4*	
	250	n.s	
*CDR2*	10	+ 5.7 ± 0.6*	Multidrug efflux transporter
	50	+ 8.5 ± 2.9*	
	125	+ 5.2 ± 2.4*	
	250	+ 3.4 ± 0.1*	

*n.s* not significant.

*p < 0.05.

**Table 2:** Genes that are downregulated in *C. albicans* after a 2 h-incubation with AEA.

Gene	AEA conc. (μg/ml)	Fold change	Function
*HWP1*	10	n.s	A major hyphal cell wall protein that acts as an adhesin and is required for normal hyphal development and cell-to-cell adhesive functions
	50	− 2.4 ± 0.6*	
	125	− 3.6 ± 1.2*	
	250	− 2.3 ± 0.3*	
*ALS3*	10	− 1.5 ± 0.3*	Agglutinin-like protein 3 acts as an adhesin that is involved in cell–cell and cell-surface interactions of hyphae
	50	− 2.8 ± 0.6*	
	125	− 5.2 ± 1.4*	
	250	− 5.2 ± 0.6*	
*HGC1*	10	− 1.3 ± 0.1*	Hypha-specific G1 cyclin-related protein 1 that regulates the CDC28 kinase during hyphal growth
	50	− 1.8 ± 0.4*	
	125	− 3.0 ± 0.1*	
	250	− 4.0 ± 0.2*	
*RAS1*	10	− 2.0 ± 0.3*	Ras-like protein 1, a GTPase that regulates both the MAP kinase signaling pathway and the cAMP signaling pathway
	50	− 1.9 ± 0.4*	
	125	− 2.5 ± 0.6*	
	250	− 4.5 ± 0.3*	
*EFG1*	10	n.s	Enhanced filamentous growth protein 1 is a basic helix-loop helix transcriptional transcription factor that regulates the switch between the white and opaque states
	50	− 2.4 ± 0.5*	
	125	− 1.8 ± 0.4*	
	250	− 1.7 ± 0.4*	
*ZAP1*	10	− 3.7 ± 0.5*	Zinc-response transcription factor that activates zinc acquisition genes that are necessary for proliferation
	50	− 4.2 ± 1.1*	
	125	− 2.4 ± 0.5*	
	250	− 5.2 ± 0.6*	
*CSH1*	10	− 2.0 ± 0.2*	Cell surface hydrophobicity-associated protein
	50	− 3.3 ± 0.8*	
	125	− 2.5 ± 0.6*	
	250	− 14.2 ± 2.6*	
*ECE1*	10	− 1.8 ± 0.3*	Extent of cell elongation protein 1 is involved in biofilm formation
	50	− 2.4 ± 0.5*	
	125	− 2.2 ± 0.8*	
	250	− 2.6 ± 0.3*	
*PLD1*	10	− 1.5 ± 0.1*	Phospholipase
	50	− 2.1 ± 0.4*	
	125	− 3.2 ± 0.7*	
	250	− 6.3 ± 1.2*	

*n.s* not significant.

*p < 0.05.

**Table 3:** Genes that are not significantly affected in *C. albicans* after a 2 h-incubation with AEA.

Function	Gene
*EAP1*	Cell wall adhesin EAP1, Cell wall protein which mediates cell–cell and cell-substrate adhesion. Required for biofilm formation and plays a role in virulence
*YWP1*	Yeast-form wall Protein 1, Cell wall protein which plays an anti-adhesive role and promotes dispersal of yeast forms, which allows the organism to seek new sites for colonization
*FKS1*	Beta-1,3-glucan synthase catalytic subunit 1
*GSP1*	GTP-binding nuclear protein GSP1/ran
*CDC35*	Adenylyl cyclase
*CST20*	Serine/threonine-protein kinase, MAP4K, required for hyphal formation and virulence
*HST7*	Serine/threonine-protein kinase
*CPH1*	Transcription factor involved in the formation of pseudohyphae and hyphae
*EFB1*	Elongation factor 1-beta
*UME6*	Transcription factor regulating filamentous growth
*TUP1*	Transcription corepressor that represses filamentous growth
*EED1*	A key regulator of hyphal maintenance

## Discussion

Candidiasis is a major health problem where *Candida* species forms biofilm on endothelial and epithelial cells. In immunosuppressed people it can lead to systemic infection and even death^6,22^. The oral cavity, the genitourinary tract and the intestine are the most frequent infection sites. It is important to find treatments that can interfere with the early adhesion of the fungi to the host cells. Here we have shown that treatment of *C. albicans* with either AEA or AraS strongly reduced their adherence to cervical epithelial cells, making them potential drugs in the co-treatment of this infectious disease. Not only do these compounds affect the hyphal attachment to epithelial cells, but they also lead to a strong reduction in the hyphal length in comparison to control. The appearance of shorter hyphae in the AEA and AraS-treated samples is a direct result of their inhibitory effect on hyphal growth. These compounds were also shown to prevent the yeast-hypha transition. Since the hyphae are associated with higher infectivity than the yeast form^6,22^, the perturbation of hyphal growth by AEA and AraS might be beneficial in reducing the virulence of *C. albicans*. Of note, AEA didn't affect the biofilm formation on polystyrene plastic surface, suggesting different requirements for the two modes of adhesion.

In order to gain better insight into the action mechanism of AEA, we undertook a gene expression study focusing on genes relevant to adhesion, biofilm formation and hyphal morphogenesis. We found genes that were upregulated by AEA, others that were down-regulated and even others that were not significantly affected. Of the genes whose expression was altered by AEA, the upregulation of *ALS1*, *TEC1* and *NRG1* and the downregulation of *HWP1*, *ALS3*, *HGC1*, *RAS1*, *ZAP1*, *CSH1*, *ECE1* and *PLD1* were the most outstanding. Als1, Als3 and Hwp1 are adhesins that are involved in the attachment of *C. albicans* to epithelial cells^7,12,23–26^. Eap1 and Als1 are important for the initial attachment to a surface, while Hpw1 and Als3 are important for the stable attachment to epithelial cells^2,6^. *EAP1* expression was unaffected by AEA, while *ALS1* was only upregulated at the lower AEA concentrations (10–50 μg/ml). The upregulation of *ALS1* showed a similar pattern to that of the transcription factor *TEC1*, which is known to regulate *ALS1* expression^6^. In contrast, *HPW1* and *ALS3* were downregulated at the higher AEA concentrations (50–250 μg/ml), an effect that seems to outweigh the upregulation of *ALS1*. This conclusion is based on the observation that *C. albicans* strains lacking *HWP1* are unable to form stable attachments to human buccal epithelial cells^23^, and specific antibodies to Als3 blocks *C. albicans* adhesion to vascular endothelial cells and buccal epithelial cells^24^. The downregulation of *HWP1* and *ALS3* together with the simultaneous downregulation of *ECE1*, which is also known to support adhesion^26^, might explain, at least partly, the reduced adhesion of AEA-treated *C. albicans* to epithelial cells.

Interesting is the AEA-mediated downregulation of *RAS1*, an upstream regulator of the Cdc35/cAMP/PKA/Efg1 and the Cdc24/Cst20/Hst7/Cph1 MAPK signal transduction pathways that regulate hyphal morphogenesis^6,22^. Ras1 is considered a master hyphal regulator and mutant *RAS1* strains show severe defects in hyphal growth^27^ and reduced adherence to epithelial cells^12^. Alteration in *RAS1* levels by AEA has thereby direct influence on hyphae formation and adhesion to epithelial cells. In addition, AEA reduced the gene expression of hyphal-promoting transcription factor *EFG1* whose activity is affected by the Ras1/Cdc35/cAMP/PKA pathway, while it had no significant effect on the expression of the hyphal-promoting transcription factor *CPH1* that is affected by the Ras1/Cdc24/Cst20/Hst7 MAPK pathway. Efg1 has been shown to be required for the adhesion of fungi to both reconstituted human epidermis and reconstituted intestinal epithelium^28^ and the cAMP/PKA/Efg1 signal transduction pathway has been demonstrated to be necessary for all stages of oral *C. albicans* infection^12^. Efg1 is an upstream regulator of the adhesins *ALS1*, *ALS3*, *ECE1* and *HWP1*^29^. The gene expression of the intermediate mediators *CDC35*, *CST20*, *HST7* were, however, unaffected by AEA. Since the signals transmitted by Ras1 are reduced by AEA, the activity of these intermediate mediators as well as the activity of the transcription factors Efg1 and Chp1 will consequently be dampened, resulting in retarded hyphal morphogenesis. The retardation of hyphae growth might further be effectuated by the prominent downregulation of the hypha-specific G1 cyclin-related protein 1 (*HGC1*) that regulates the Cdc28 kinase during hyphal growth^30^. On top of these effects, the AEA-mediated upregulation of *NRG1*, a transcriptional repressor of filamentous growth^31^, may further contribute to the observed reduction in hyphae length and size. Nrg3 has been shown to repress the expression of *ALS3*^29^, *ECE1*^31^ and *HPW1*^31^. Thus the upregulation of *NRG1* together with the downregulation of *EFG1* may fortify the repression of the adhesin genes. Moreover, the AEA-mediated downregulation of the zinc-responsive transcription factor *ZAP1* that is known to be required for efficient hyphae formation^32,33^, might have an additional impact. Altogether, the combined alterations in gene expression caused by AEA might explain both the AEA-induced inhibition of hyphal growth and the reduced adherence of AEA-treated hyphae to epithelial cells.

AEA and 2-AG were originally discovered to be endocannabinoids that bind to the cannabinoid receptor CB1^34^. It later emerged that AEA has anti-anxiety activities by regulating the neurotransmitter system by being a retrograde synaptic messenger^35,36^. AEA binds also to other membrane molecules in mammalian besides CB1 including CB2, GRP55 (CB3) and TRPV1^36^. AEA usually does not bind to the extracellular part of the receptors, but rather, its binding domain is frequently located deep in the plasma membrane^37^. The interactions of AEA with membrane-bound cholesterol and ceramides facilitate its transport to the receptor binding domains^37^. The binding of AEA to TRPV1 leads to transient calcium influx in neurons^38^. It remains to be determined whether AEA also affects calcium influx in fungi.

In conclusion, AEA and AraS prevent yeast-hyphae transition, inhibit hyphal growth and reduce the ability of *C. albicans* hyphae to adhere to epithelial cells. This is, among others, achieved by altered expression of genes involved in cell–cell interaction and of genes regulating hyphal morphogenesis.

## Material and methods

### Chemicals

Anandamide (AEA), *N*-arachidonoyl serine (AraS) and 2-arachidonoylglycerol (2-AG) were synthesized as described^39–41^ and dissolved in ethanol. We also purchased anandamide from Sigma (St. Louis, MO), and *N*-arachidonoyl serine (AraS) and 2-arachidonoylglycerol (2-AG) from Cayman Chemical.

### Cell lines

HeLa cervical carcinoma cells were cultivated in DMEM (Sigma, St. Louis, MO) supplemented with 8% heat-inactivated fetal calf serum (FCS; Biological Industries, Beth HaEmek, Israel), 2 mM L-glutamine and 1 mM sodium pyruvate, and incubated at 37 °C in a humidified atmosphere containing 5% CO_2_.

### Fungal strain and growth conditions

*C. albicans* SC5314 that has the GFP gene integrated within the ENO1 genomic locus^42^, was kindly provided by Prof. J. Berman (Tel Aviv University, Israel). The fungi were first seeded on potato-dextrose agar plates (Acumedia, Neogen, Lansing, MI) at room temperature where they grow in the yeast form, and then inoculated in RPMI (Sigma, St. Louis, MO) for a 16–18 h incubation at 37 °C to let them form hyphae. For the yeast-hypha transition assay, colonies of *C. albicans* in yeast form were inoculated in RPMI at an OD_600nm_ of 0.5 and incubated with different concentrations of AEA, AraS and 2-AG at 37 °C for 4 h. For time-lapse microscopy, hyphae that has been formed after 4 h incubation of *C. albicans* in yeast form (OD_600nm_ of 0.25) at 37 °C, were incubated in 300 μl RPMI in a μ-slide 8 well chambered coverslip (ibidi GmbH, Martinsried, Germany) in the absence or presence of 125 μg/ml AEA. The Okolab incubation chamber was used to maintain the temperature at 37 °C. Images were captured each 5 min for 3 h using the Nikon spinning disk microscope (Yokogawa W1), the × 20 CFI PLAN APO VC objective and the SCMOS ZYLA camera. The images were processed using the NIS-Element AR program.

### Biofilm formation on plastic culture plates

*C. albicans* that have been cultivated overnight in RPMI at 37 °C, were resuspended and diluted to an OD_600nm_ of 0.25 in RPMI and then seeded in 96-flat bottomed microplates (Corning, NY) in 200 μl RPMI with different concentrations of test compounds per well. Following an overnight incubation at 37 °C, the biofilms formed in the wells were washed twice with PBS and stained for 20 min with 1% crystal violet. The stained biofilms were washed twice with DDW and after drying, the stain was dissolved in 200 μl of 33% acetic acid and the absorbance read in a Tecan M200 microplate reader at 595nm^20^.

### *C. albicans* adherence to HeLa cells

The adherence assay was performed by a slight modification of Feldman et al.^43^. Two hundred and fifty thousand HeLa cells were seeded in 1 ml DMEM supplemented with 8% FCS in 24 well tissue culture plate (Corning, NY) the day before assay. At the following morning, the medium was changed to 1 ml of RPMI supplemented with 1% FCS. *C. albicans* that have grown for 16–18 h at 37 °C, were resuspended in fresh RPMI to an OD of 1.0, and then exposed to different concentrations of AEA, 2-AG, AraS or corresponding concentrations of ethanol for 1 h serving as controls. Thereafter, 100 μl of the pretreated *C. albicans* were added to the 1 ml HeLa cell cultures, and the co-culture incubated for 1 h at 37 °C. At the end of incubation, the cells were washed twice with 1 ml PBS and fixed with 1% paraformaldehyde (Electron Microscopy Sciences, Hatfield, PA) in PBS for 30 min. The co-cultures were visualized using NIKON confocal microscope and the NIS-Element AR software. Quantitative analysis of GFP was done using the ImageJ software. For each sample 8–10 images were taken and each image was analyzed for the amount of GFP. The percentage adherence was calculated according to relative GFP staining. In addition, the length of the hyphae was measured using the Adobe Photoshop software. Between 50 and 100 fungi were measured per sample.

### Real-time quantitative PCR

*C. albicans* that have been grown overnight in RPMI at 37 °C were treated for 2 h with various concentrations of AEA or corresponding concentrations of ethanol. At the end of incubation, the RNA was extracted from the fungi using TRI-Reagent (Sigma, St. Louis, MO)^44^. Cell disruption was done in 1 ml TRI-Reagent in the presence of 200 μl 1 mm acid-washed glass beads (Sigma, St. Louis, MO) with the aid of a FastPrep cell disrupter (BIO 101, Savant Instrument). The purified RNA was reverse transcribed into cDNA using the qScript cDNA synthesis kit (Quantabio, Beverly, MA) and PCR amplification was done in a CFX96 BioRad Connect Real-Time PCR apparatus using Power Sybr Green Master Mix (Applied Biosystems, ThermoFischer Scientific) on 2 ng cDNA in the presence of 300 nM forward/reverse primer sets (Table 4). PCR conditions included an initial heating at 50 °C for 2 min, an activation step at 95 °C for 10 min, followed by 40 cycles of amplification (95 °C for 15 s, 60 °C for 1 min). Calculations were done according to the 2^−ΔΔCt^ method, where both 18S rRNA and ACT1 were used as reference genes.

**Table 4:** Primers used for real-time PCR.

Gene	Forward primer	Reverse primer
*18S rRNA*	TCTTCTTGATTTTGTGGGTGG	TCGATAGTCCCTCTAAGAAGTG
*ACT1*	AAGAATTGATTTGGCTGGTAGAGA	TGGCAGAAGATTGAGAAGAAGTTT
*ALS1*	TTGGGTTGGTCCTTAGATGG	ATGATTTCAAAGCGTCGTTC
*ALS3*	TAATGCTGCTACGTATAATT	CCTGAAATTGACATGTAGCA
*CDC35*	TTCATCAGGGGTTATTTCAC	CTCTATCAACCCGCCATTTC
*CDR1*	GTACTATCCATCAACCATCAGCACTT	GCCGTTCTTCCACCTTTTTGTA
*CDR2*	TGCTGAACCGACAGACTCAGTT	AAGAGATTGCCAATTGTCCCATA
*CPH1*	ATGCAACACTATTTATACCTC	CGGATATTGTTGATGATGATA
*CSH1*	CTGTCGGTACTATGAGATTG	GATGAATAAACCCAACAACT
*CST20*	TTCTGACTTCAAAGACATCAT	AATGTCTATTTCTGGTGGTG
*EAP1*	AGGCAAAGGTGGCTATCAAG	GTGCAGTCGTGTAGGAGGT
*ECE1*	GCTGGTATCATTGCTGATAT	TTCGATGGATTGTTGAACAC
*EED1*	AGCAACGACTTCCAAAAGGA	CGGTTTCTGGTTCGATGATT
*EFB1*	GCTGCTAAAGGTCCAAAACC	CATCCCATGGTTTGACATCC
*EFG1*	TATGCCCCAGCAAACAACTG	TTGTTGTCCTGCTGTCTGTC
*FKS1*	CGTGAAATTGATCATGCCTGTAC	AACCCTTCTGGGCTCCAAA
*GSP1*	TGAAGTCCATCCATTAGGAT	ATCTCTATGCCAGTTTGGAA
*HGC1*	AATTGAGGACCTTTTGAATGGAAA	AAAGCTGTGATTAAATCGGTTTTGA
*HST7*	ACTCCAACATCCAATATAACA	TTGATTGACGTTCAATGAAGA
*HWP1*	CACAGGTAGACGGTCAAGGT	AAGGTTCTTCCTGCTGTTGT
*MDR1*	TCAGTCCGATGTCAGAAAATGC	GCAGTGGGAATTTGTAGTATGACAA
*NRG1*	CCAAGTACCTCCACCAGCAT	GGGAGTTGGCAGTAAATCA
*PLD1*	GCCAAGAGAGCAAGGGTTAGCA	CGGATTCGTCATCCATTTCTCC
*RAS1*	GGCCATGAGAGAACAATATA	GTCTTTCCATTTCTAAATCAC
*TEC1*	AGGTTCCCTGGTTTAAGTG	ACTGGTATGTGTGGGTGAT
*TUP1*	CTTGGAGTTGGCCCATAGAA	TGGTGCCACAATCTGTTGTT
*UME6*	AGCACCAAATTCGCCTTATG	AGGTTGAGCTTGCTGCAGTT
*YWP1*	GCTACTGCTACTGGTGCTA	AACGGTGGTTTCTTGAC
*ZAP1*	ATCTGTCCAGTGTTGTTTGTA	AGGTCTCTTTGAAAGTTGTG

### Statistical analysis

Experiments were performed in triplicates and repeated twice. Results are presented as average of data obtained from three experiments ± standard error. Results are considered to be statistically significant when the *p* value was less than 0.05 using the Student's t-test.

## Supplementary information

Supplementary Information

Supplementary Figure 3a

Supplementary Figure 3b

## Abbreviations

AEA: Anandamide, *N*-arachidonoylethanolamine
2-AG: 2-Arachidonoylglycerol
ALS1: Agglutinin-like sequence protein 1
ALS3: Agglutinin-like sequence protein 3
AraS: *N*-Arachidonoyl serine
BCR1: Biofilm and cell wall regulator 1
CDC35: Cell-division-cycle gene 35
CPH1: *Candida* pseudohyphal regulator 1*,* a homolog of STE12-like transcription factor
CSH1: Cell surface hydrophobicity associated protein 1
CST20: *C. albicans* homologous to the p21-activated kinase (PAK) kinase Ste20, a serine/threonine protein kinase
CZF1: *C. albicans* Zinc finger protein, a transcription factor
EAP1: Enhanced adherence to polystyrene 1
ECE1: Extent of cell elongation protein 1
EED1: Epithelial escape and dissemination 1, a transcriptional regulator
EFB1: Elongation factor 1-beta.
EFG1: Enhanced filamentous growth protein 1
FKS1: FK506 sensitivity, a 1,3-beta-glucan synthase
GSP1: Genetic suppressor of Prp20-1, a GTP-binding nuclear protein
HGC1: Hypha-specific G1 cyclin-related protein 1
HST7: Homologous to the MAPK kinase (MAPKK) Ste7, a serine/threonine protein kinase
HWP1: Hyphal wall protein 1
MDR1: Multidrug resistance protein 1
CDR1: Candida drug resistance 1, an ATP-dependent multidrug transporter 1
CDR2: Candida drug resistance 2, an ATP-dependent multidrug transporter 2
MKC1: Mitogen-activate protein kinase from *C. albicans*
NRG1: Negative regulator of glucose-repressed genes 1, a transcriptional regulator
PGA34: Predicted GPI-anchored protein 34
PLD1: Phospholipase D1
RAS1: Ras-like protein 1
TEC1: Transposon enhancement control, a TEA/ATTS transcription factor
TUP1: dTMP-uptake, a chromatin-silencing transcriptional regulator
UME6: Unscheduled meiotic gene expression, a transcriptional regulator
YWP1: Yeast-form wall protein 1
ZAP1: Zinc-responsive activator protein 1
