# Supplementary material for: Anandamide prevents the adhesion of filamentous Candida albicans to cervical epithelial cells
Source: Sci Rep. 2020 Aug 13;10:13728. doi: 10.1038/s41598-020-70650-6 (PMC7426432; doi:10.1038/s41598-020-70650-6)
Supplement: Supplementary file 1 — Supplementary Information [file 41598_2020_70650_MOESM1_ESM.pptx]

## Slide 1
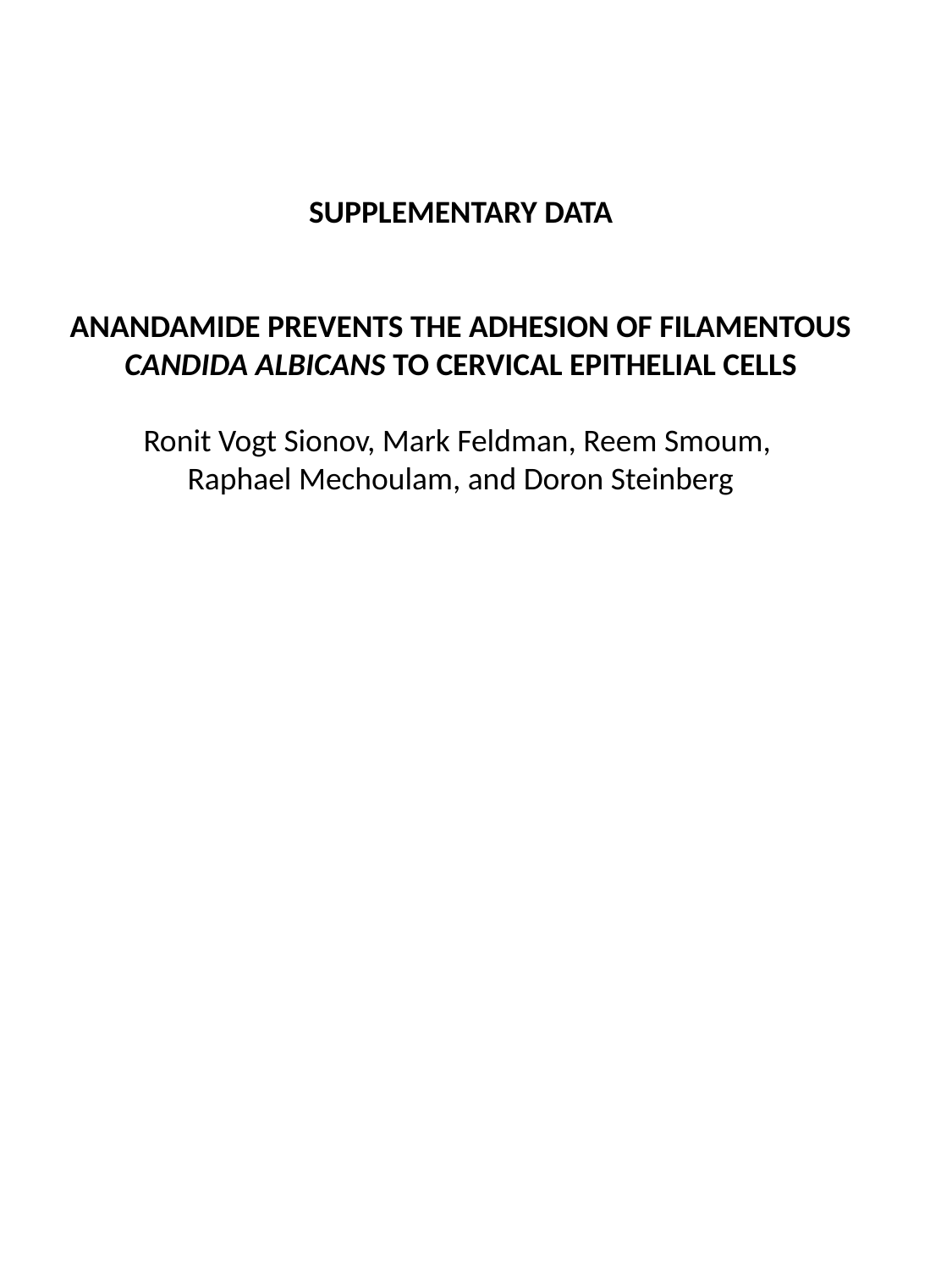

SUPPLEMENTARY DATA
ANANDAMIDE PREVENTS THE ADHESION OF FILAMENTOUS CANDIDA ALBICANS TO CERVICAL EPITHELIAL CELLS
Ronit Vogt Sionov, Mark Feldman, Reem Smoum,
Raphael Mechoulam, and Doron Steinberg

## Slide 2
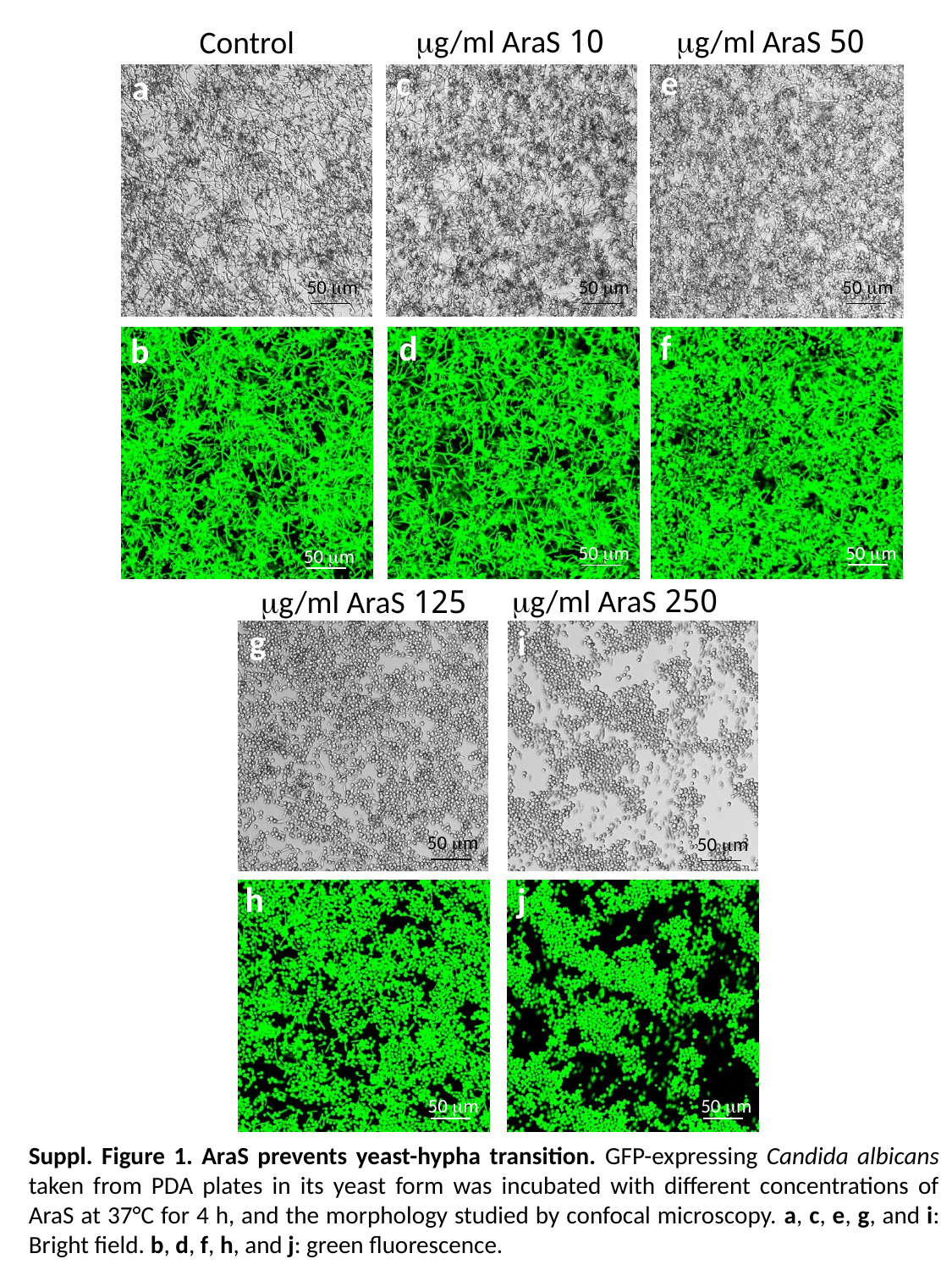

50 mg/ml AraS
10 mg/ml AraS
Control
e
c
a
50 mm
50 mm
50 mm
f
d
b
50 mm
50 mm
50 mm
250 mg/ml AraS
125 mg/ml AraS
g
i
h
j
50 mm
50 mm
50 mm
50 mm
Suppl. Figure 1. AraS prevents yeast-hypha transition. GFP-expressing Candida albicans taken from PDA plates in its yeast form was incubated with different concentrations of AraS at 37°C for 4 h, and the morphology studied by confocal microscopy. a, c, e, g, and i: Bright field. b, d, f, h, and j: green fluorescence.

## Slide 3
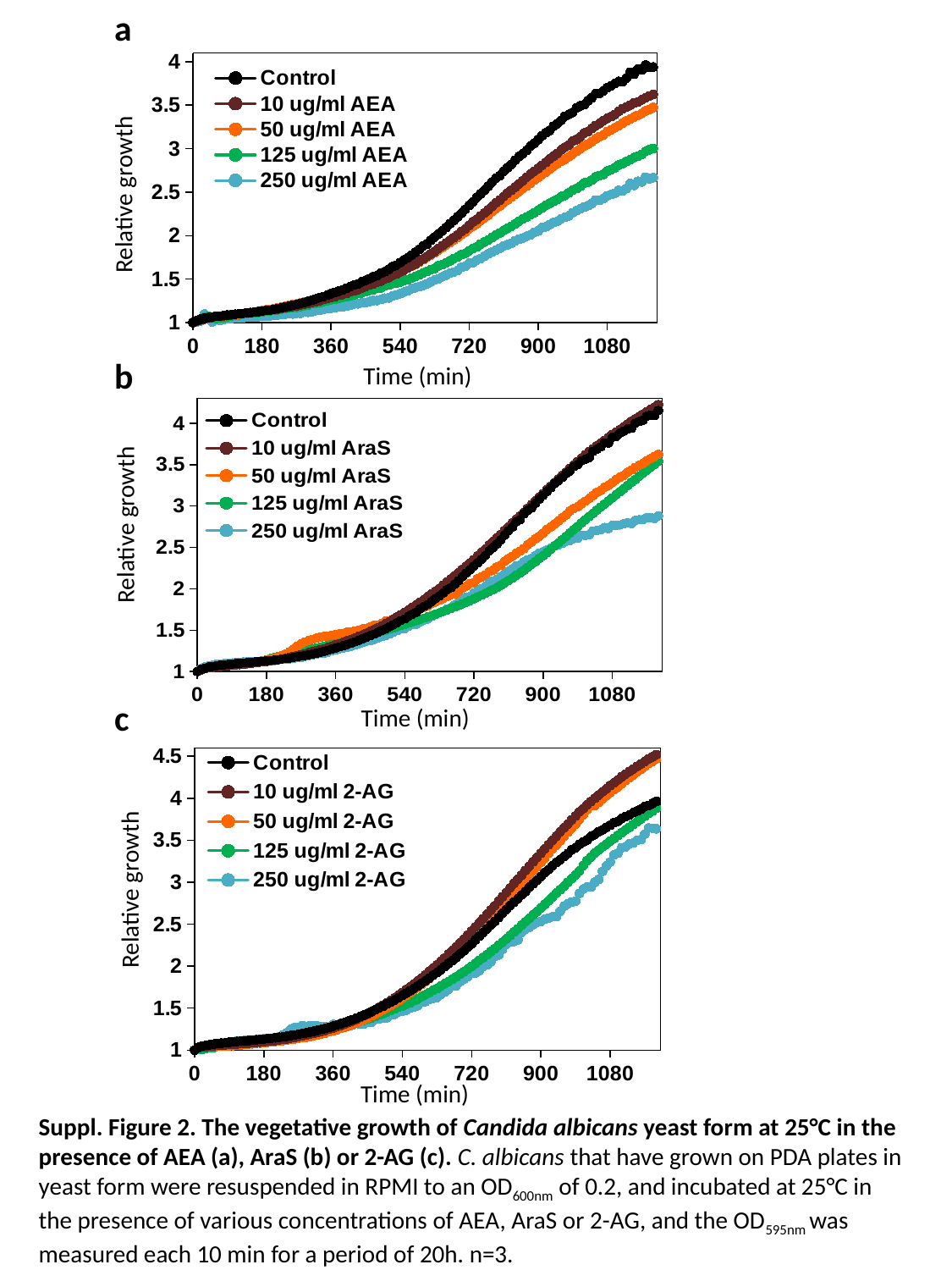

a
### Chart
| Category | Control | 10 ug/ml AEA | 50 ug/ml AEA | 125 ug/ml AEA | 250 ug/ml AEA |
|---|---|---|---|---|---|Relative growth
b
Time (min)
### Chart
| Category | Control | 10 ug/ml AraS | 50 ug/ml AraS | 125 ug/ml AraS | 250 ug/ml AraS |
|---|---|---|---|---|---|Relative growth
c
Time (min)
### Chart
| Category | Control | 10 ug/ml 2-AG | 50 ug/ml 2-AG | 125 ug/ml 2-AG | 250 ug/ml 2-AG |
|---|---|---|---|---|---|Relative growth
Time (min)
Suppl. Figure 2. The vegetative growth of Candida albicans yeast form at 25°C in the presence of AEA (a), AraS (b) or 2-AG (c). C. albicans that have grown on PDA plates in yeast form were resuspended in RPMI to an OD600nm of 0.2, and incubated at 25°C in the presence of various concentrations of AEA, AraS or 2-AG, and the OD595nm was measured each 10 min for a period of 20h. n=3.

## Slide 4
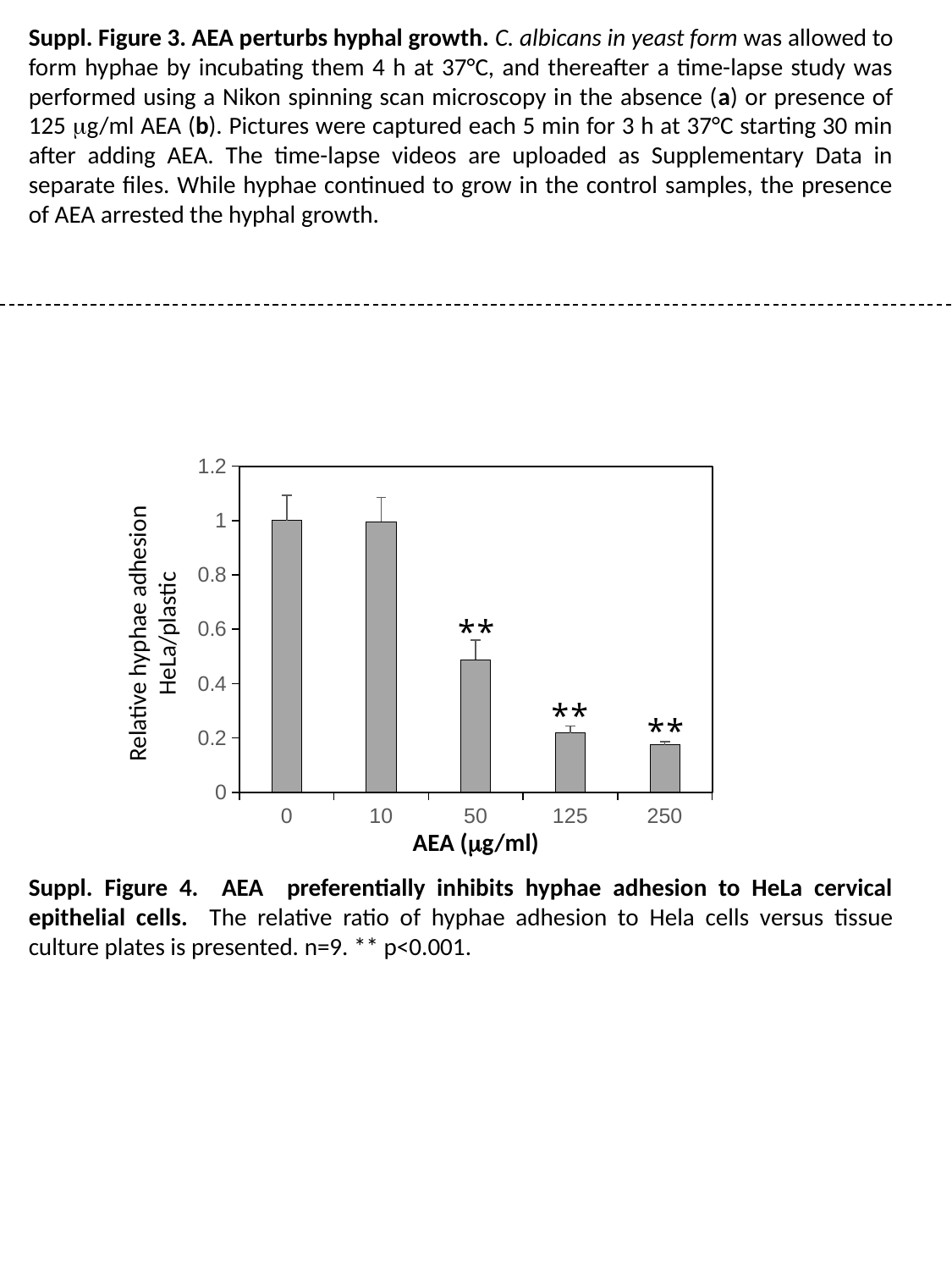

Suppl. Figure 3. AEA perturbs hyphal growth. C. albicans in yeast form was allowed to form hyphae by incubating them 4 h at 37°C, and thereafter a time-lapse study was performed using a Nikon spinning scan microscopy in the absence (a) or presence of 125 mg/ml AEA (b). Pictures were captured each 5 min for 3 h at 37°C starting 30 min after adding AEA. The time-lapse videos are uploaded as Supplementary Data in separate files. While hyphae continued to grow in the control samples, the presence of AEA arrested the hyphal growth.
### Chart
| Category | |
|---|---|
| 0 | 1.0 |
| 10 | 0.9955273501450581 |
| 50 | 0.4882606597157174 |
| 125 | 0.21778073314510146 |
| 250 | 0.17470796159780574 |Relative hyphae adhesion
HeLa/plastic
**
**
**
AEA (mg/ml)
Suppl. Figure 4. AEA preferentially inhibits hyphae adhesion to HeLa cervical epithelial cells. The relative ratio of hyphae adhesion to Hela cells versus tissue culture plates is presented. n=9. ** p<0.001.

## Slide 5
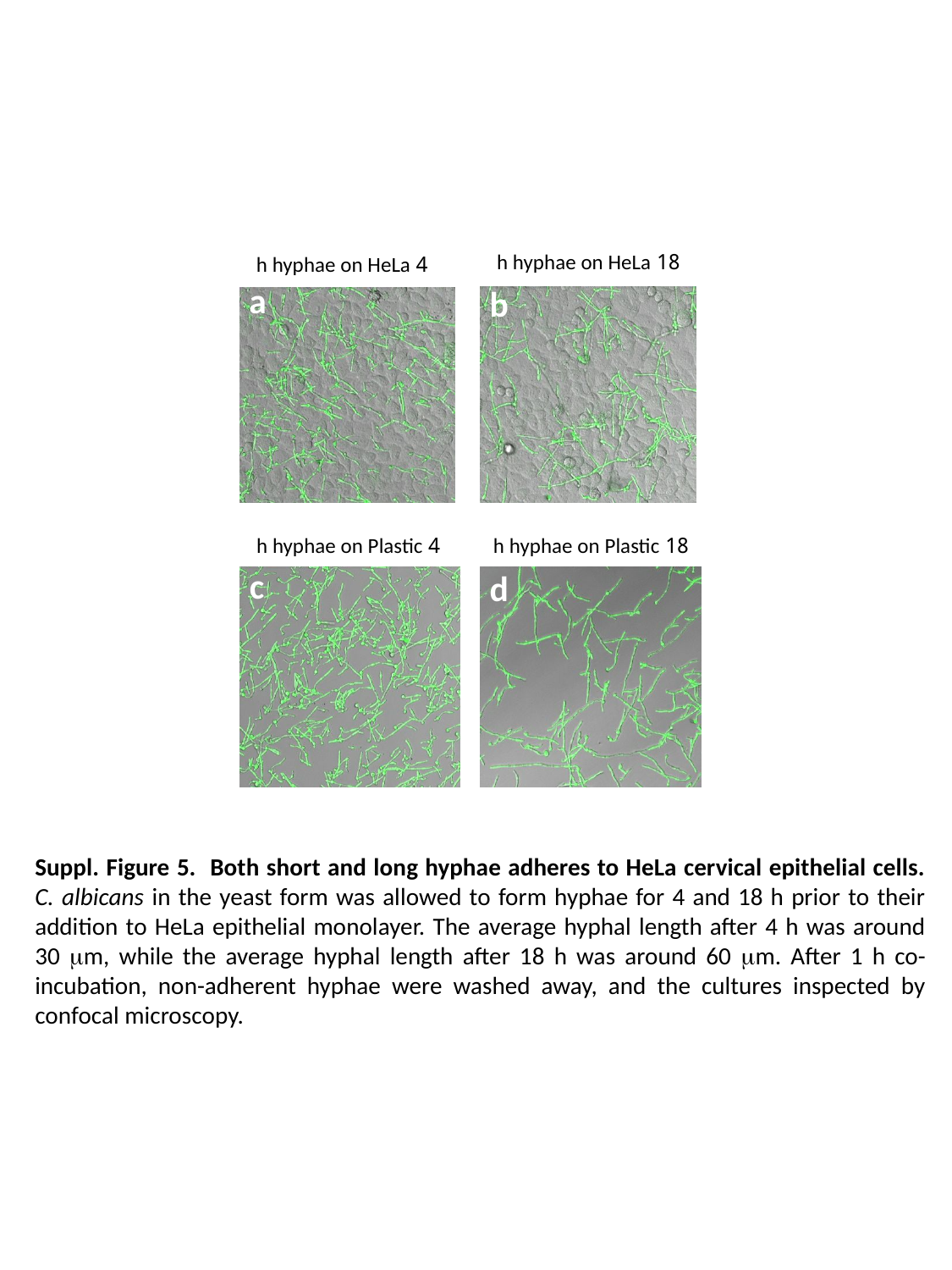

18 h hyphae on HeLa
4 h hyphae on HeLa
4 h hyphae on Plastic
18 h hyphae on Plastic
a
b
c
d
Suppl. Figure 5. Both short and long hyphae adheres to HeLa cervical epithelial cells. C. albicans in the yeast form was allowed to form hyphae for 4 and 18 h prior to their addition to HeLa epithelial monolayer. The average hyphal length after 4 h was around 30 mm, while the average hyphal length after 18 h was around 60 mm. After 1 h co-incubation, non-adherent hyphae were washed away, and the cultures inspected by confocal microscopy.

## Slide 6
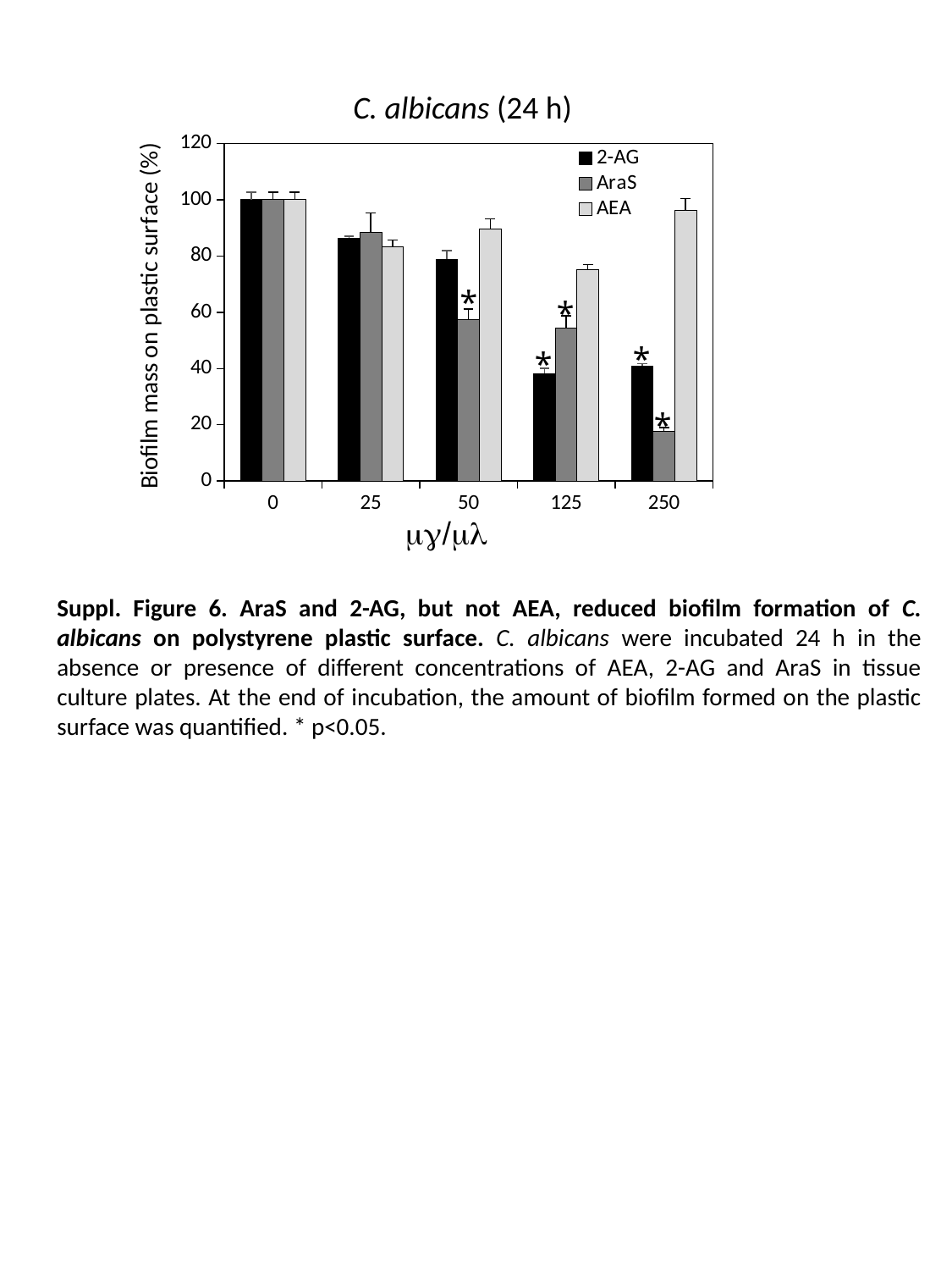

C. albicans (24 h)
### Chart
| Category | 2-AG | AraS | AEA |
|---|---|---|---|
| 0 | 100.0 | 100.0 | 100.0 |
| 25 | 86.36902830979002 | 88.40101354451141 | 83.34668823037588 |
| 50 | 78.85324375755432 | 57.347522401219976 | 89.46187918903831 |
| 125 | 38.18884163926388 | 54.474871290605265 | 75.19599968241558 |
| 250 | 40.91580552067286 | 17.679809434898615 | 96.29966514799698 |*
*
*
*
*
Suppl. Figure 6. AraS and 2-AG, but not AEA, reduced biofilm formation of C. albicans on polystyrene plastic surface. C. albicans were incubated 24 h in the absence or presence of different concentrations of AEA, 2-AG and AraS in tissue culture plates. At the end of incubation, the amount of biofilm formed on the plastic surface was quantified. * p<0.05.
